# Evolutionary diversification of Japanese *Stomaphis* aphids (Aphididae, Lachninae) in relation to their host plant use and ant association

**DOI:** 10.1007/s00114-020-1671-4

**Published:** 2020-03-19

**Authors:** Tetsuya Yamamoto, Mitsuru Hattori, Yoshiyuki Matsumoto, Shouhei Ueda, Takao Itino

**Affiliations:** 1 Interdisciplinary Graduate School of Science and Technology, Shinshu University, Nagano, Japan; 2 Graduate School of Fisheries and Environmental Sciences, Nagasaki University, Nagasaki, Japan; 3 Shibaura Institute of Technology Kashiwa Junior and Senior High School, Chiba, Japan; 4 Graduate School of Life and Environmental Science, Osaka Prefecture University, Osaka, Japan; 5 Department of Biology, Faculty of Science, Shinshu University, Nagano, Japan

**Keywords:** Host shift, Phylogenetic reconstruction, Phytophagous insect, Species specificity

## Abstract

Phytophagous insects are among the most diverse of the earth’s organisms, and their diversification patterns and the driving forces behind these have attracted considerable research interest. Host shifting to closely related plant species is thought to play an important role in phytophagous insect diversification, but the extent to which other interactions such as mutualistic associations affect diversification is not yet known. In this study, we reconstructed the molecular phylogeny of Japanese *Stomaphis* aphids and determined whether host shifting or mutualistic association with different ant species could explain diversification in this aphid genus. We analyzed 12 species of *Stomaphis* and grouped them into ten well-supported DNA lineages. Species in each lineage used a single or a few host plant species, but were mutualistically associated with many ant species of the genus *Lasius*. This result suggests that *Stomaphis* evolutionarily diversified primarily through host plant shifts. Interestingly, the reconstructed phylogeny suggests that *Stomaphis* host shifts occasionally occurred between very distantly related host plant taxa (spanning up to five plant orders). The dependence of *Stomaphis* on long-lasting *Lasius* ant colonies situated in temperate deciduous forests where *Lasius* is the dominant ant genus may have led the aphids to shift to distantly related but spatially adjacent host tree species.

**Electronic supplementary material:**

The online version of this article (10.1007/s00114-020-1671-4) contains supplementary material, which is available to authorized users.

## Introduction

One of the goals of evolutionary biology is to understand the factors and mechanisms that have led to species diversity on the earth. Insects are among the most diverse taxa described thus far, and phytophagous insects account for more than 40% of all described insects (Grimaldi and Engel 2005). Therefore, the patterns and driving forces of their diversification have been studied extensively (Agosta 2006; Futuyma and Agrawal 2009; Depa et al. 2017).

Phytophagous insects are hypothesized to have diversified by ecological speciation through adaptation to different, often closely related host plant species, followed by the interruption of gene flow owing to disruptive selection. Many studies have provided evidence supporting this hypothesis, both by reconstructing phylogenies of phytophagous insects to examine the evolutionary diversification of their host plant use (Funk et al. 1995; Peccoud et al. 2009) and by comparing host preference and performance (e.g., growth rate) among host lineages (Nosil et al. 2002; Matsubayashi et al. 2011, 2013; Fujiyama et al. 2013). For example, Peccoud et al. (2009) assessed clonal lineages of the pea aphid, *Acyrthosiphon pisum*, and found eight sympatric host lineages, each of which was specialized to a different host plant species.

Interspecific interactions other than those with host plants can also drive the diversification of phytophagous insects (Pierce et al. 2002). For example, Depa et al. (2017) reported that two sister aphid species diverged because of their association with different ant species. Phytophagous insect diversification through interactions with organisms other than host plants is still poorly understood; however, the relative importance of host plants and other organisms in structuring insect evolutionary diversification needs to be elucidated.

Phytophagous aphids are highly specific to host plants; 99% of aphid species use a single specific plant species or a few closely related plant species (Blackman and Eastop 1994; Dixon 1998). Furthermore, even aphid species that appear to be “generalists” may be genetically differentiated into different host lineages, as in the case of *A. pisum* mentioned above (Peccoud et al. 2009). Therefore, aphid diversity has generally been attributed to adaptive diversification to different closely related host plant species (Dixon 1998; Drès and Mallet 2002).

Aphids also establish mutualistic relationships with ants by providing them with honeydew in return for protection from natural enemies and hygienic services (Hölldobler and Wilson 1990; Stadler and Dixon 2005), and some aphid traits have evolved owing to the selection pressure from attending ants: for example, an anal plate shaped to hold honeydew (Heie 1987; Kanturski et al. 2017), a long proboscis and stylet (Shingleton et al. 2005), or a particular cuticular hydrocarbon profile (Lang and Menzel 2011). In addition, some aphid traits are plastically induced by the presence of attending ants: for example, production of more and higher-quality honeydew (Fischer and Shingleton 2001) or a smaller flight apparatus (Yao 2012). These adaptations and the fact that different ant species offer different degrees of protection to the aphids with which they associate (Novgorodova 2005) suggest that adaptive diversification to particular ant species might occur.

The genus *Stomaphis* is a group of large aphids having about 4–7 mm of body length; they also have a long proboscis and stylet, which, in adult females, may be up to twice the body length (Brożek et al. 2015). *Stomaphis* aphids use this long mouthpart to suck phloem sap from tree trunks. Thirty-three species and four subspecies of *Stomaphis* have been described worldwide, and most have been described as specific to a single plant species or genus (Blackman and Eastop 2019). However, the morphological classification of *Stomaphis* may not accurately reflect the phylogenetic relationships within the genus *Stomaphis*; for example, Depa et al. (2012) reported discrepancies between the molecular phylogeny of some *Stomaphis* species and their morphological classification.

Globally, host plants used by aphids of the genus *Stomaphis* belong to 13 families in seven orders, although most aphid species usually specialize to a single or few closely related plant species (Blackman and Eastop 2019). This huge taxonomic breadth of host plant usage suggests that *Stomaphis* aphids may have diversified through host plant shifts, occasionally between very different taxa. Indeed, European *Stomaphis* species comprise two sister mtDNA lineages, each of which uses a different and distantly related host plant species (Sapindales and Malpighiales; Depa and Mróz 2013), suggesting that diversification occurred by a host plant shift.

Importantly, *Stomaphis* aphids interact with not only plants but also ants, with which they have a mutualistic relationship. The long mouthparts of *Stomaphis* aphids restrict their mobility, rendering it difficult for them to escape from their natural enemies. Because *Stomaphis* aphids strongly depend on attending ants for protection from predators and receiving hygienic services, they cannot survive without ants (Lorenz and Scheurer 1998). In at least one case, aphid diversification resulted from such aphid–ant interaction; Depa et al. (2017) reported that the sister species *Stomaphis quercus* and *S. wojciechowskii* share the same host plant species, but each maintains a mutualistic relationship with a different ant species. Each aphid species has evolved morphological and ecological characteristics suitable for interaction with its own partner ant species. For example, a population of *S. quercus* associated with ant species that is a partner of *S. wojciechowskii* acquires morphological characters same as those of *S. wojciechowskii*. Such ant-related diversification in *Stomaphis* aphids suggests that aphid–ant mutualism can lead to the diversification of these phytophagous insects. However, this phenomenon has been recognized in only these two *Stomaphis* species, and whether this mode of diversification occurs more generally in this genus is not yet known.

This study aimed to elucidate the influence of interactions with host plants and associated ants on phylogenetic diversification in the genus *Stomaphis.* First, we reconstructed the phylogeny of *Stomaphis* species in Japan from mitochondrial and nuclear DNA sequences and then investigated the relationships between the phylogenetic lineages and host plant utilization and ant association. The results suggested that diversification in Japanese *Stomaphis* aphids occurred through interactions with host plants rather than with associated ants.

## Methods

### Field sampling

We discovered 160 *Stomaphis* aphid colonies at 34 sites in Japan by searching for known host plants and/or by following *Lasius* ant trails (Table S1). We considered all aphids on a single host plant to belong to a single colony. The aphids and associated ants were collected from each host plant and stored in 99.5% and 70% ethanol, respectively, at 4 °C before DNA extraction and morphological identification.

### Interspecific relationships

To clarify the correspondence between aphid phylogeny and aphid interspecific interactions, we identified all host plants and attending ant species. Host plants were identified on the basis of leaf and stem morphology, and associated ant species were identified on the basis of their mitochondrial *COI* sequences, because it is often difficult to identify *Lasius* ants at the species level on the basis of morphology alone. Protocols and primers for mitochondrial *COI* sequence analyses of *Lasius* ants were referred from Maruyama et al. (2008).

### DNA extraction and sequencing of aphids

Total genomic DNA was extracted from a single aphid by using a DNeasy Blood & Tissue Kit (Qiagen) following manufacturer’s instructions. We targeted two molecular markers—mitochondrial cytochrome oxidase c subunit II (*COII*) and exon of nuclear elongation factor 1α (*EF-1α*). The *COII* gene was amplified using polymerase chain reaction (PCR) analysis and Takara Tks Gflex DNA polymerase (Takara Bio, Shiga, Japan) by using the PCR primer set mt2993+ (5′-CATTCATATTCAGAATTACC-3′) and Eva-R (5′-GAGACCATTACTTGCTTTCAGTCATCT-3′; Brower and Jeansonne 2004; Stern 1994). The *EF-1α* gene was amplified using PCR with Takara Ex Taq DNA polymerase (Takara Bio, Shiga, Japan) by using the PCR primer set efs175 (5′-GGAAATGGGAAAAGGCTCCTTCAAGTAYGCYTGGG-3′) and efa1082 (5′-ATGTGAGCAGTGTGGCAATCCAA-3′; Normark 1999). The PCR temperature profile was 30 cycles at 98 °C for 10 s, 50 °C for 10 s, and 72 °C for 60 s for COII and 30 cycles at 98 °C for 10 s, 42 °C for 30 s, and 72 °C for 60 s for *EF-1α*. After amplification, the PCR product was purified using ExoSap-IT reagent (USB; Cleveland, OH, USA). Cycle sequencing reactions for both strands were performed using a BigDye Terminator version 1.1 Cycle Sequencing Kit (ABI, Weiterstadt, Germany) on an ABI 3130 Genetic Analyzer.

### Phylogenetic analyses

The mitochondrial *COII* and *EF-1α* sequences of 589 bp (*COII*) and 723 bp (*EF-1α*) were edited and aligned using the SeqScape v. 2.5 software (ABI; Weiterstadt, Germany). We selected the best-fit substitution model by using Bayesian information criterion 4 (BIC4) in a Kakusan4 software package (Tanabe 2007): for *COII*, we used J2 + G for the first and second codon positions and J1 + G for the third codon position; for *EF-1α*, we used HKY85 + G for the first and third codon positions and JC69 + H for the second codon position. We performed a maximum likelihood analysis by using TREEFINDER version October 2008 software (Jobb et al. 2004) and the substitution models selected above. Clade support was assessed using 1000 bootstrap replications by using TREEFINDER. The mitochondrial *COII* genetic distance was calculated using Kimura 2-Parameter (K2P) model by using Mega7 (Kumar et al. 2016). Next, we identified host plants and attending ants associated with each reconstructed phylogenetic lineage. The mitochondrial (*COII*) haplotype network estimated using the median-joining network (MJ) method was constructed using PopART ver. 1.7 (Leigh and Bryant 2015). To facilitate the understanding of the relationship between *Stomaphis* aphids and host plants, we reconstructed the character of host plant use on a haplotype network.

### Morphological identification

To identify aphid species on the basis of morphology, we collected apterous viviparous or oviparous adult females from one or few *Stomaphis* colonies belonging to each phylogenetic lineage or (if members of the lineage used more than one host plant species) from each host plant species. Each sample was immersed in 10% KOH and encapsulated in a Canada balsam by using the method of Kozarzhevskaya (1986). We identified morphological species by referencing to taxonomic and biological traits (Inouye 1938; Takahashi 1960; Sorin 1965, 1979, 1995) and by measuring each part of the aphid’s body under an optical microscope following the key to Japanese *Stomaphis* species (Sorin 2012). All slide samples are now in Matsumoto’s collection.

## Results

### Morphological identification

For measuring morphological features of *Stomaphis* aphids, 12 morphological species (*S. abieticola*, *S. aceris*, *S. aphananthae*, *S. fagi*, *S. hirukawai*, *S. japonica*, *S. malloti*, *S. matsumotoi*, *S. pterocaryae*, *S. takahashii*, *S. ulmicola*, and *S. yanonis*), two subspecies (*S. pini takaoensis* and *S. yanonis aesculi*), and three undescribed species (*Stomaphis* spp. 1–3) were identified (Table S1). Of the 15 species and three subspecies described in Japan, three species (*S. alni*, *S. carpini*, and *S. pini*) and one subspecies (*S. asiphon sakuratanii*) were not available.

### Phylogeny of *Stomaphis* aphids

**Fig. 1:**
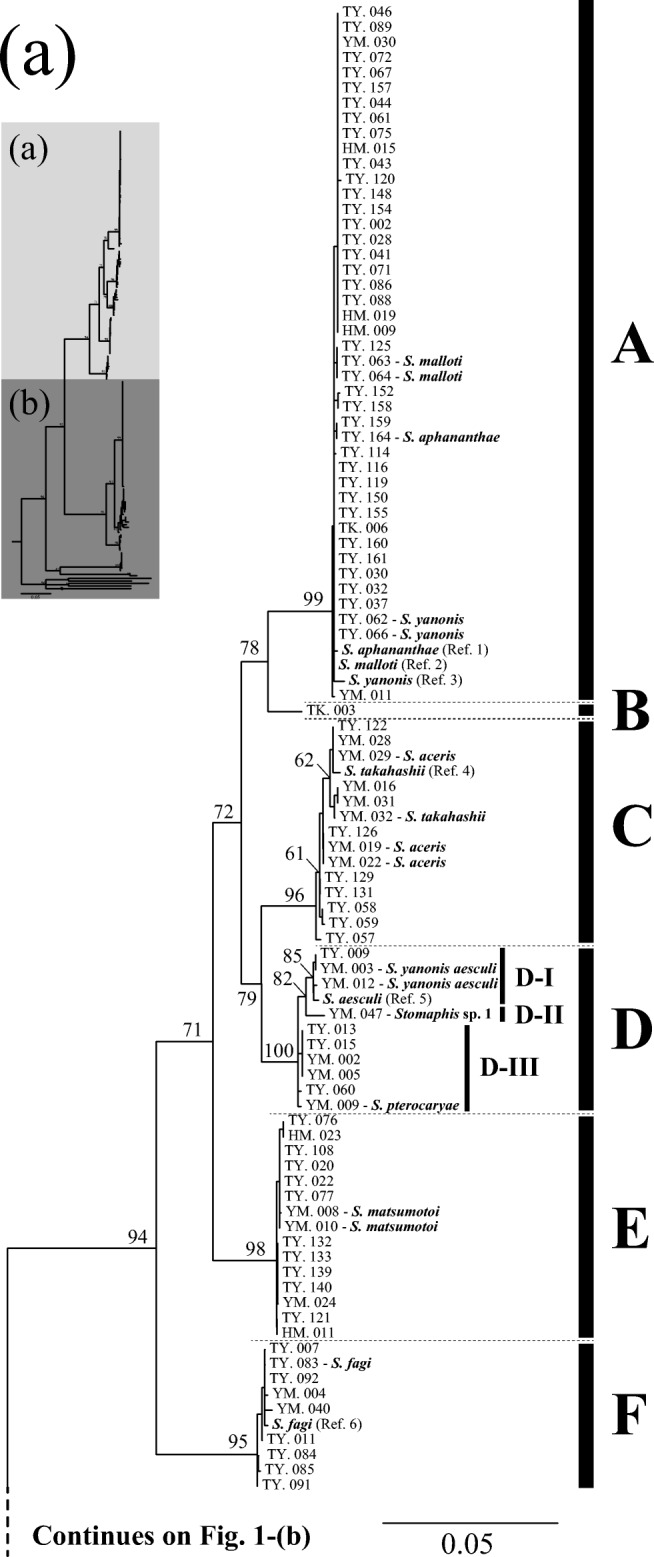

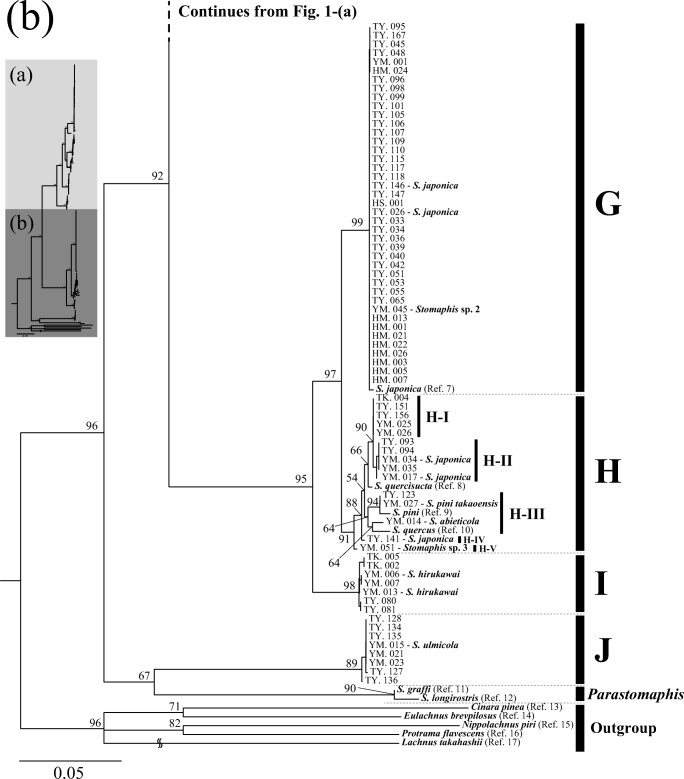
Maximum likelihood phylogenetic tree of *Stomaphis* aphid samples based on mitochondrial *COII* and nuclear *EF-1α* sequences. The inset at the top left shows an overview of the complete tree and the parts shown in **a** and **b**. The tree shows ten major lineages (A to J). Lineages D and H each comprise several sublineages (D-1 to D-3, H-1 to H-5). The sample number and species, either morphologically identified or referenced from GenBank (if known), are shown for each operational taxonomic unit. See Table S1 for details of the samples. The bootstrap probability is shown for each node, and the scale indicates a nucleotide substitution rate of 0.05

The combined sequence matrix used for phylogenetic reconstruction was 1312 bp long. The collected Japanese *Stomaphis* specimens were grouped into ten major DNA lineages (A to J) with a COII genetic distance by K2P model greater than 0.03 (Fig. 1). Each DNA lineage was supported by a ML bootstrap value with a probability of more than 70% and included one or more previously described species: lineage A (*S. aphananthae*, *S. malloti*, and *S. yanonis*); lineage B (samples for morphological identification could not be obtained); lineage C (*S. aceris* and *S. takahashii*); lineage D (*S. pterocaryae*, *S. yanonis aesculi*, and *Stomaphis* sp. 1); lineage E (*S. matsumotoi*); lineage F (*S. fagi*); lineage G (*S. japonica* and *Stomaphis* sp. 2); lineage H (*S. abieticola*, *S. japonica*, *S. pini takaoensis*, and *Stomaphis* sp. 3); lineage I (*S. hirukawai*); and lineage J (*S. ulmicola*). Lineages D and H were subdivided into three and five sublineages, respectively, according to host plant usage; each sublineage utilizes a different host plant species (Fig. 1). Because lineage B consisted of a single sample, we refrain from discussing its relationship to plants and ants.

### Host plant use

**Fig. 2:**
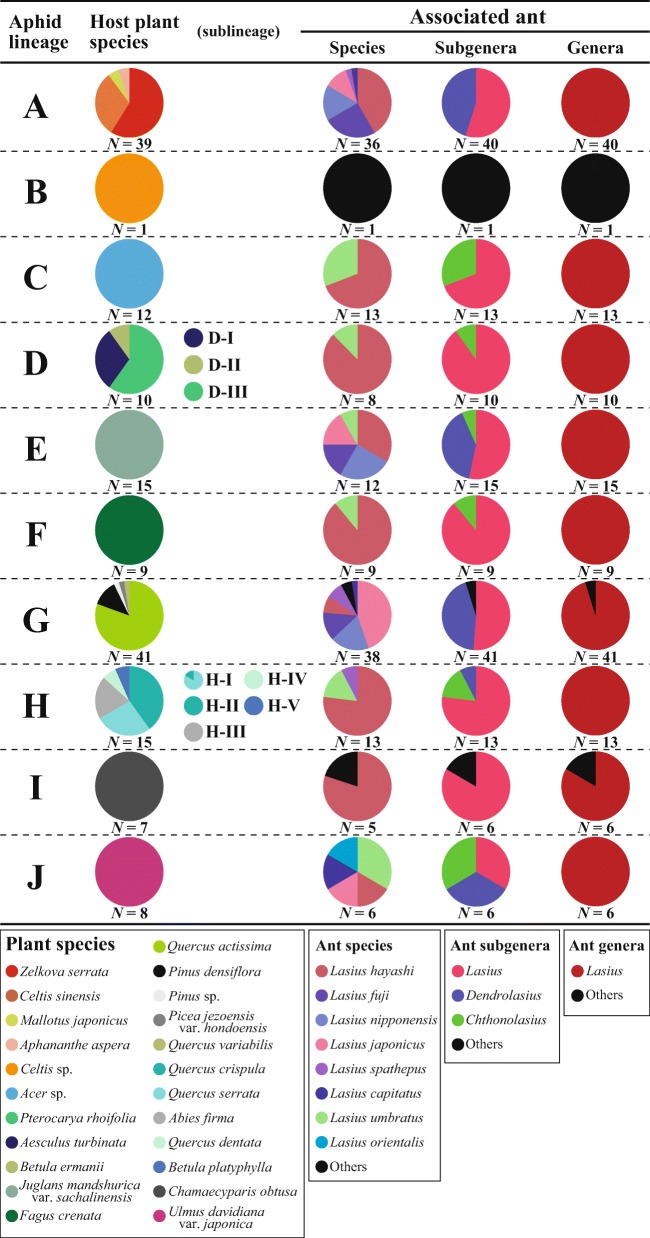
Specificity of *Stomaphis* lineages to host plant and associated ant species. Letters in the left column indicate the DNA lineages inferred by molecular phylogenetic analysis by using mitochondrial and nuclear DNA sequences (Fig. 1). The pie charts show the compositions of host plant species and associated ant species and subgenera and genera for each aphid lineage (see the Supporting information, Tables S2 and S3, for the complete data set). For lineages D and H, a host plant pie chart is also shown for each sublineage. The number of samples (number of aphid colonies for which the host plant or associated ant were identified) *N* is shown below each pie chart

Each of the five *Stomaphis* aphid lineages (C, E, F, I, and J) used a single plant species as a host, whereas each of the other four lineages (A, D, G, and H) used three or more host plant species (Fig. 2, Table S2). Each sublineage in lineages D and H (D-I, D-II, D-III, H-I, H-II, H-III, H-IV, and H-V), except H-I, used a single host plant species. Host plant species mostly did not overlap among the lineages or sublineages, except between sublineages H-I and H-II. In all, the *Stomaphis* aphids used 22 host plant species belonging to 15 genera, ten families, and five orders, and the evolutionary host plant shift associated with aphid speciation was overwhelmingly wide (between orders; Table S2). To our knowledge, this is the first study to document *Betula ermanii*, *Picea jezoensis* var. *hondoensis*, *Quercus crispula*, and *Q. dentata* as host plant species of *Stomaphis* aphids in Japan (Inouye 1938; Takahashi 1960; Sorin 1965, 1979, 1995, 2012).

### Haplotype analysis

**Fig. 3:**
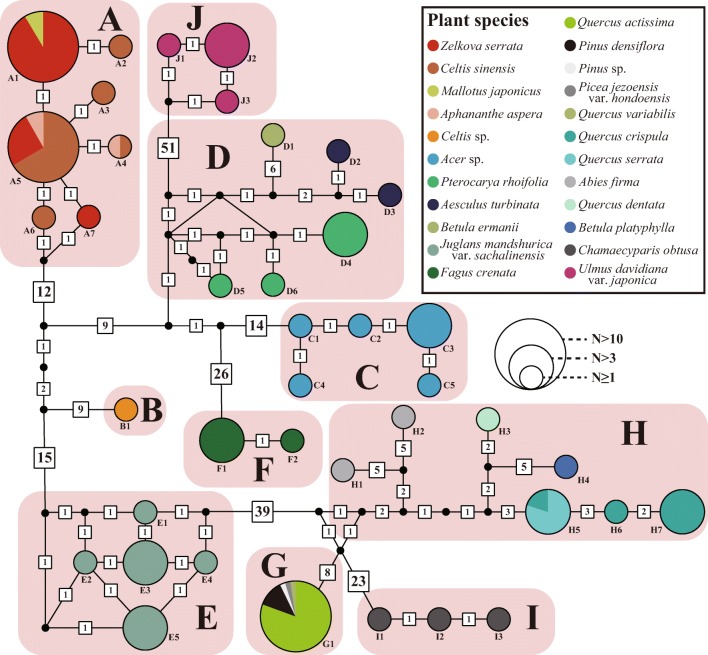
Mitochondrial haplotype network of *Stomaphis* aphids. Numbers near the circle indicate haplotype numbers (see Table S1). The size of the circle indicates the number of samples of the haplotype. The color of the circle indicates the proportion of plants used by the haplotype. The number in the box indicates the number of mutations

From the 160 *COII* sequences of *Stomaphis* aphids, a total of 38 haplotypes were identified (Fig. 3). When the lineages of each sample determined using molecular phylogenetic analysis was reconstructed in the haplotype network, each sample was integrated in the same group as the lineages in the phylogenetic analysis (e.g., haplotype group A corresponds to lineage A in molecular phylogenetic analysis).

Some haplotypes were detected in haplotype groups C, E, F, I, and J, and all haplotypes in these groups used single plant species. In haplotype group A, seven haplotypes were detected. The haplotypes A1, A4, and A5 used multiple plant species. Conversely, haplotypes A2, A3, A6, and A7 used single plant species, but these plant species overlapped with other haplotypes in group A. In haplotype group D, six haplotypes were detected. Each haplotype uses a single plant species. In haplotype group G, one haplotype using multiple plant species was detected. In haplotype group H, seven haplotypes were detected. Except for haplotype H5, each haplotype used a single plant species. Haplotype H5 used two plant species, one of which is the same species used by haplotypes H6 and H7.

### Mutualistic association with ants

All of the investigated *Stomaphis* aphid colonies were attended by ant workers. Ants of genus *Lasius* were the most frequent (97%; 150/154 aphid colonies; Tables S2 and S3). Considering their *COI* nucleotide sequences, eight *Lasius* ant species belonging to three subgenera (*Lasius*, *Dendrolasius*, and *Chthonolasius*) were identified. Other observed attending ant species were *Camponotus obscuripes*, *Crematogaster* sp., and *Polyrhachis lamellidens* (Table S1).

The ant subgenus *Lasius* accounted for 61% (95/154 aphid colonies) of all associated ants, and it was the most frequent subgenus among ants associated with all *Stomaphis* aphid lineages except lineage J (*N* = 6; Fig. 2). The ant subgenus *Dendrolasius* accounted for 29% (45/154) of all associated ants, and ants of this subgenus attended aphids of five *Stomaphis* lineages (A, E, G, H, and J). The ant subgenus *Chthonolasius* accounted for 7% (11/154) of all associated ants and attended aphids of six *Stomaphis* lineages (C, D, E, F, H, and J).

## Discussion

Phylogenetic analysis of *Stomaphis* aphids and their relationships with host plants and attending ants revealed that each lineage and haplotype of *Stomaphis* aphids showed a high degree of specificity to host plant species, and no species-level host plant overlap was noted among lineages. Conversely, almost all lineages of *Stomaphis* aphids were associated with two or more ant species. These findings suggest that *Stomaphis* evolved and diversified owing to host plant shifts, whereas diversification rarely followed associated ant shifts. In addition, most *Stomaphis* aphid lineages were associated exclusively with ants of the genus *Lasius*, indicating that the mutualism between *Stomaphis* and *Lasius* has been very tight.

### Species-specific host plant use in *Stomaphis*

Most of the DNA lineages and haplotypes of *Stomaphis* aphids used a single plant species as host (Figs. 2 and 3). In general, aphids have high specificity to their host plants because of the need to adapt and specialize to plant species-specific traits such as nutrient composition, defense systems (external morphology and secondary metabolites), and phenology (Dixon 1998; Peccoud et al. 2010). *Stomaphis* aphids can also benefit by adapting physiologically, morphologically, and ecologically to plant species-specific traits. Thus, like other phytophagous insects (War et al. 2012), *Stomaphis* aphids may exhibit a pattern of specificity for a particular plant species.

### Evolution of host plant use in *Stomaphis*

*Stomaphis* aphids use a phylogenetically broad range of host plants. In Japan, their host plants belong to ten families in five orders (Table S2); globally, their host plants belong to 13 families in seven orders (Blackman and Eastop 2019). A conspicuous result of this study is that, in Japan, host plant shifts of *Stomaphis* aphids have occurred between taxa that are widely separated phylogenetically. Phylogenetic constraints on host plant utilization by aphids usually occur, and many aphid genera or families are associated strictly with a single plant genus or family (Peccoud et al. 2010). Because plant phenology, chemical compounds, and nutritional value are similar among closely related host plant species (Prasad et al. 2012; Davies et al. 2013), host shifts by aphids may occur only between plants belonging to, for example, a single genus. For example, conifer-feeding aphids of the genus *Cinara*, belonging to the same subfamily, Lachninae, as the genus *Stomaphis*, comprise as many as 250 species worldwide, all of which use host plants belonging to three families (Pinaceae, Cupressaceae, and Taxaceae) in the order Pinales (Blackman and Eastop 2019). Thus, the phylogenetic breadth of host plants used by the *Stomaphis* aphids in our study is clearly different from the general pattern of aphid diversification. Moreover, this host plant use pattern probably does not reflect the extinction of intermediate lineages, because relatively closely related aphid lineages, such as the sublineages in D and H, show host shifts to distantly related plant taxa. For example, within lineage D, the sublineages reflect host shifts from *Pterocarya* and *Betula* (Fagales) to *Aesculus* (Sapindales; Fig. 1 and Table S2).

Similar evolutionary host shifting by phloem sap feeders has been shown in the treehopper *Enchenopa binotata* species complex; each host lineage in this complex is specific to a single plant species, but together the host lineages use various plant taxa (Wood and Guttman 1983). Wood and Guttman (1983) inferred that this pattern reflects, first, fidelity to a single plant species, which arises because specialization on a particular plant species is advantageous for each host lineage; second, a release from phylogenetic constraints that enables the occurrence of shifts between distantly related plant species (Wood and Keese 1990; Wood 1993; Hsu et al. 2018).

As noted above, specializing physiologically, morphologically, and ecologically to a single host plant species is advantageous for *Stomaphis* aphids. Subsequently, they may release from phylogenetic constraints such as nutrient value, defense systems, and phenology of plant species. In lineage H, host shift was noted between angiosperm (*Abies*) and gymnosperm (*Quercus*) trees. The nutrient value, secondary metabolite composition, and bark morphology differed between the two groups. For example, *Abies* tree has the resin composed of terpenes, which may be assumed to be considerably toxic for insects, and *Stomaphis* aphids must overcome this toxic resin when they use *Abies* tree as their host. Therefore, the acquisition of novel host plants for *Stomaphis* aphids may not be restricted strongly by plant physiological and morphological traits. In addition, because the tree trunk stores large amounts of nutrients throughout the season, *Stomaphis* aphids can use the phloem sap and survive even in winter (Depa 2013; Depa et al. 2015b). That is, seasonal variations of nutrients does not affect *Stomaphis* aphids; in contrast, almost all other aphid species need to ecologically adapt to seasonal variations of nutrients (e.g., host alternation, aestivation, and galling). Therefore, when *Stomaphis* aphids switch to novel host plants, they may not be threatened by the differences of plant phenology, thereby weakening the phylogenetic constraints for the availability of plant use.

### Does strong dependence on ant mutualism affect the pattern of plant use?

*Stomaphis* aphids have a sedentary life mode, possibly because of their large body size, which is necessary for sucking phloem sap from tree trunks and consequent low dispersal ability. This sedentary life mode leads in turn to their strong dependence on ant mutualism (Depa et al. 2015a). Moreover, their sedentary life mode suggests that gene flow between aphids on different host plants would be extremely low, which would promote disruptive selection. As in the treehopper *E. binotata* species complex (Wood and Guttman 1983), a sedentary life mode owing to their dependence on ant mutualism may be one of the factors promoting disruptive selection in Homoptera by using different host plants (Wood 1982, 1987). Moreover, Depa et al. (2017) showed that more sedentary species of *Stomaphis* exhibit greater genetic variation and use a broader range of host plant taxa than less sedentary sister species found in the same area.

Depa et al. (2017) have shown that *Stomaphis* aphids can be accidentally transferred to neighboring tree species by attending ants. The results of this study also suggest that *Stomaphis* aphids might have dispersed in this manner. If transfer by attending ants occurs, then “generalist” *Stomaphis* species might use host plant species that, although distantly related taxonomically, have similar environmental preferences and are distributed sympatrically. Indeed, although the four host plant species used by *Stomaphis* lineage A belong to three different plant families, they all are found in a sunny, dry, and lowland forest edge environment. Similarly, the three host plant species used by *Stomaphis* lineage D belong to three different plant families, but grow in moist mountain forest environments. Determination of the factors that have led *Stomaphis* aphids to shift to distantly related host plants and their consequent evolutionary diversification is a topic for future studies. Such studies would allow us to gain insights into the mechanisms for the diversification of phytophagous insects.

### Evolution of mutualistic ant association in *Stomaphis*

In this study, most *Stomaphis* colonies were associated with ants of the genus *Lasius* (Fig. 2), suggesting that mutualism with *Lasius* ants is important for the survival of *Stomaphis* aphids. *Lasius* ants usually nest at the base of trees (Terayama et al. 2014); hence, *Stomaphis* aphids living on tree trunks may be more likely to encounter *Lasius* ants than ants of other genera. In addition, *Lasius* worker ants walk up tree trunks in large numbers to collect food resources high up on the tree (Terayama et al. 2014); therefore, they can easily defend aphids living on the tree trunks against their natural enemies. Moreover, *Lasius* ant colonies persist for long periods of several years or more (Matsuura and Yashiro 2006), which enhances their ability to act as a stable partner of sedentary *Stomaphis* aphids. The strong defense provided to *Stomaphis* aphids by *Lasius* ants can compensate for the aphids’ low escape ability owing to their large body and long proboscis. Future investigations of survival and reproduction rate differences between *Stomaphis* colonies associated with *Lasius* and those associated other ant taxa should provide further insight into the evolution of this aphid–ant mutualism.

A high proportion of aphid colonies in the *Stomaphis* lineages were attended by ants of the subgenus *Lasius* (Fig. 2), which are among the most common ants in Japan and occur in a wide range of environments from bare land to forest (Terayama et al. 2014). In addition, Matsuura and Yashiro (2006) reported that ants of the subgenus *Lasius* build shelters made of soil over *Stomaphis* aphid colonies on tree trunks and protect *Stomaphis* aphid eggs in their nests during the winter. These ant behaviors suggest that ants of this subgenus are among the most useful mutualistic partners for *Stomaphis*.

Aphids of several lineages were attended by ants of the subgenus *Dendrolasius* in relatively low proportion. Establishment of a mutualistic association between *Stomaphis* aphids and ants of the subgenus *Dendrolasius* might be difficult. The morphological traits of the European aphid *S. quercu*s (dark, slender, shiny body, and a strong degree of cuticle sclerotization) make them inconspicuous to natural enemies and tolerant of a harsh environment and also well adapted to *Dendrolasius* ant protection, because these ants do not build shelters over *Stomaphis* aphid colonies on tree trunks, but directly attend the aphids (Depa et al. 2017). In our survey, we found many *Dendrolasius* ant colonies in Tokamachi, Niigata Prefecture; however, although aphid colonies of lineage A (TY.089) were attended by *Dendrolasius* ants, those of lineage F observed in this area (TY.083, TY.084, TY.085, TY.091, and TY.092) were not. In addition, morphologically, *S. yanonis* (lineage A) are dark and slender, but *S. fagi* (lineage F) are white and round (Matsumoto 2008). These facts suggest that only species such as *S. yanonis* and *S. quercus*, which have acquired certain morphological traits, can associate with the ant subgenus *Dendrolasius*. In the future, investigation of the comparative morphology of many *Stomaphis* species in relation to their associated attending ant species would likely reveal the ant mutualism-related adaptations in *Stomaphis*.

Species specificity to the associated ant species has been reported in many obligate ant mutualisms (e.g., between ants and plants (Quek et al. 2004), or ants and Lycaenid butterflies (Pierce et al. 2002)). In *Stomaphis* aphids, Depa et al. (2017) reported one example where speciation was apparently driven by ant–aphid interactions. However, the *Stomaphis* lineages identified in this study were not associated with specific ant species. Endo and Itino (2012) showed that *S. yanonis* (lineage A in this study) successfully avoids attack and maintains its intimate relationship with its attending ant species, *Lasius fuji*, by having cuticular hydrocarbons similar to those of *L. fuji* worker ants. This finding suggests that *S. yanonis* is adapted to a specific attending ant species. However, colonies in lineage A were associated with not only *L. fuji* but also other ant species; one explanation may be that *S. yanonis* adapt locally to different ant species by changing their cuticular hydrocarbon, in areas where the density of *L. fuji* ants is low.

### Classification of *Stomaphis*

Until recently, aphids of the genus *Stomaphis* were classified on the basis of their morphological characteristics, although, in some cases, the morphological classification of *Stomaphis* has been modified on the basis of phylogenetic relationships reconstructed using genetic markers (Depa and Mróz 2013). The molecular phylogeny of *Stomaphis* in Japan reconstructed in this study differed in part from the morphological classification, suggesting that the classification of some Japanese species, for example, of lineages A, D, G, and H, should be revised. However, no colonies of *S. alni* and *S. carpini*, other *Stomaphis* species that have been described in Japan (Sorin 1965), were sampled in this study. Therefore, more extensive sampling and more detailed morphological and ecological information are necessary to classify accurately Japanese *Stomaphis*.

In particular, Takada (2008) conducted fragmentary observations over 10 years and indicated that *S. japonica* may alternate hosts between *Quercus serrata* as primary and *Quercus acutissima* as secondary, although such host alternation is rare in Lachninae. In this study, the aphids using *Q. serrata* and *Q. acutissima* as host belonged to different lineages (lineages G and H). *Stomaphis* aphids on *Q. acutissima* certainly alternate their hosts because they fly away from *Q. acutissima* during winter. Therefore, *Stomaphis* aphids on *Q. serrata* and *Q. acutissima* might be different species, and aphids using *Q. acutissima* might have different primary host plants. Although a previous study was conducted in Kyoto (Takada 2008), we could not cover this area. Further investigation of the morphology and life history of lineages G and H in a wide area is needed to reveal the host alternation in *Stomaphis*.

## Electronic supplementary material

ESM 1 (XLSX 40 kb)
